# Antitubercular evaluation of root extract and isolated phytochemicals from *Lophira lanceolata* against two resistant strains of *Mycobacterium tuberculosis*

**DOI:** 10.1080/13880209.2018.1476559

**Published:** 2018-07-03

**Authors:** Jeanne Louise Nkot, Dominique Serge Ngono Bikobo, Auguste Abouem A Zintchem, Norbert Mbabi Nyemeck, Esther Del Florence Moni Ndedi, Patrick Hervé Betote Diboué, Dieudonné Emmanuel Pegnyemb, Christian G. Bochet, Ulrich Koert

**Affiliations:** a Department of Organic Chemistry, Faculty of Science, University of Yaoundé I, Yaoundé, Cameroon;; b Department Chemie, Universität Fribourg, Fribourg, Switzerland;; c Department of Chemistry, Higher Training College University of Yaoundé I, Yaoundé, Cameroon;; d Faculty of Chemistry, Philipps-Universität Marburg, Marburg, Germany;; e Department of Microbiology, Faculty of Science, University of Yaoundé I, Yaoundé, Cameroon

**Keywords:** Ochnaceae, dihydrolophirone A, biflavonoids, steroids, antitubercular activity

## Abstract

**Context:** The roots of *Lophira lanceolata* Van Tiegh. Ex Keay (Ochnaceae) have numerous medicinal values in the Central African region. Even though the MeOH extract of the roots has shown antimycobacterial activities, the constituents responsible for this inhibitory activity remain unknown.

**Objective:** Phytochemical investigation of the MeOH root extract of *L. lanceolata* and determination of the antimycobacterial activities of that extract and constituents against the growth of *Mycobacterium tuberculosis*.

**Materials and methods:** Column chromatography was used to provide bioactive phytoconstituents. Those compounds were elucidated using MS and NMR spectroscopic data. Antimycobacterial screening of the extract (4.882–5000 µg/mL in DMSO during 24 h at 37 °C) and isolated compounds (0.244–250 µg/mL in DMSO during 24 h at 37 °C) was performed by microplate alamar blue assay (MABA) against two mycobacterial strains.

**Results:** The investigation of *L. lanceolata* MeOH roots extract provided of mixture of unseparated biflavonoids with a newly described one, dihydrolophirone A (**1a**) associated to lophirone A (**1b**). The bioactive compounds that effectively inhibited the growth of *M. tuberculosis* AC45 were found to be compounds **1** and **2.** They exhibited MIC values of 31.25 and 15.75 µg/mL, respectively, and their MIC was found to be 62.5 µg/mL against resistant strain AC83.

**Discussion and conclusions:** It is clearly evident from the results obtained that the mycobacterial activity of *L. lanceolata* could be related mainly to its steroid and flavonoid contents. Therefore, this study suggests the potential of the above-mentioned classes of compounds as promising candidate agents for developing new anti-tuberculosis drugs.

## Introduction

Tuberculosis (TB) is a chronic contagious and deadly disease that spreads through the air. The number of patients infected with the disease is rising worldwide. In 2015, there were an estimated 10.4 million cases of TB with over 400,000 deaths resulting from TB disease among people living with HIV (World Health Organization [WHO] 2016). The proportion of TB cases living with HIV was highest in the African region despite the implementation of the standardized control strategy (Directly Observed Treatment Short course [DOTS]) (WHO 1996). Thus, the incidence, prevalence and mortality rates of TB in Africa have continued to be on increase (WHO 2016). In addition, the high susceptibility of human immunodeficiency virus-infected persons to this illness, the proliferation of HIV/AIDS, and the emergence of multidrug-resistant strains of *Mycobacterium tuberculosis* (MDR) are contributing to the worsening impact of this disease (Bloom 2002). Currently, the first-line regimen for treating TB is considered old and prescribes rifampicin (RMP) and isoniazid (INH) as component drugs. These antibiotics overtime affect the rise of multi-drug resistant (MDR). This increasing drug resistance incidence has led to an urgent need to develop new antitubercular drugs with low toxicity to overcome these limitations and increase the armamentarium of the existing therapeutic drugs.

The genus *Lophira* (Ochnaceae) is found throughout tropical regions of Africa (Ghogomu et al. 1989a). The genus is widely exploited commercially to build houses and to make furniture (Tih et al. 1992). *Lophira lanceolata* Van Tiegh. Ex Keay is a tall tree reaching up to 60 feet high growing in the woody savannah forests. This plant is used in African folk medicine for the treatment of diseases related mainly to toothache, liver infections, female sterility, cough, fever, heart pains, blood spitting, intercostal pain, stomach pain, dysmenorrhea, respiratory troubles, and to relieve the gripping of dysentery (Persinos et al. 1967; Ghogomu et al. 1989a). Previous phytochemical analyses of the genus *Lophira* have resulted in the isolation of biflavonoids and tetraflavonoids (Ghogomu et al. 1987, 1989a, 1989b, 1990; Tih et al. 1992; Pegnyemb et al. 1994; Sani et al. 2010, 2011; Tih, Ghogomu, et al. 2003; Tih, Martin, et al. 2003), nitrile glycosides (Murakami et al. 1993; Tih et al. 1994), benzamides (Persinos et al. 1967), a benzoylglucoside (Pegnyemb et al. 1998), triterpenoids (Sani et al. 2011).

In continuation of our phytochemical studies on *Lophira lanceolata*, other chemical constituents of the roots of this species have been investigated; newly isolated compounds could support the traditional uses of the plant, based on their medicinal interest. Although there is no information on the antitubercular activity of this plant or its constituents, previous reports on antitubercular activity of steroids and biflavonoids have designated immense prospects in this field (Camacho-Corona et al. 2009; Thakur and Gothwal 2015). It has been claimed that several plant natural products inhibit several species of mycobacteria (Okunade et al. 2004; Pauli et al. 2005). The *Lophira* genus through the richness of its components could be considered as an important source for new antitubercular agents.

We report herein the isolation and identification of a new biflavonoid derivative and other constituents from the methanol extract of *Lophira lanceolata* with their antitubercular properties.

## Materials and methods

### General procedures

Melting points were uncorrected and were measured on a Mettler Toledo instrument. IR spectra were recorded on an Alpha FT-IR Spectrometer from Bruker, while 1D and 2D NMR spectra were obtained on a Bruker DRX 500 (500 MHz for ^1^H and 125 MHz for ^13^C spectra) spectrometer (Bruker, Rheinstetten, Germany) with chemical shifts reported in *δ* (ppm) using TMS (*δ*_H_) as an internal standard. The HR-ESI-MS were obtained on LTQ-FT instrument (Thermo Scientific, Waltham, MA). UPLC–MS were measured by a Shimadzu UPLC–MS system using a *L*^−^ column 2 ODS (I.D. 2.1 × 100 mm, Chemical Evaluation and Research Institute, Tokyo, Japan), at a flow rate of 0.2 mL/min, a detection wavelength of 350 and 300 nm, and FMW (HCOOH/MeCN/H_2_O = 1:12:87) as eluent, ESI^+^ 4.5 kV, ESI^−^ 3.5 kV, 250 °C. Optical rotations were measured on a Perkin-Elmer 341 polarimeter. Silica gel 60 (230–400 mesh E. Merck, Darmstadt, Germany) was employed for column chromatography (CC), the solvent mixing systems for elution were mainly CH_2_Cl_2_–MeOH with increasing polarity. Analytical thin layer chromatography (TLC) was carried out on Merck silica gel (Merck, Darmstadt, Germany) 60 PF_254_ plates (0.25 mm layer thickness).

### Plant material

The stem roots of *L. lanceolata* were collected at Balamba, near Yaoundé in the Center region of Cameroon (4° 26′. 00′′ N, 11° 14′. 00′′ E) in July 2014 and authenticated by Mr. Victor Nana a botanist. A voucher specimen (N° 45596 HCN) was deposited at the National Herbarium in Yaoundé, Cameroon.

### Extraction and isolation

Dried and powdered stem roots of *L. lanceolata* (854 g) was extracted with methanol (6.0 L, three times) at room temperature. The combined methanol extract was evaporated under reduced pressure to give a thick gummy mass (34.6 g) that was suspended in water and successively extracted with *n*-hexane, dichloromethane and ethyl acetate to afford the corresponding subfractions. The ethyl acetate soluble sub-fraction (13.0 g) was subjected to CC (4 × 120 cm; 150 g of silica gel 230–400 mesh) eluting with a gradient solvent system (CH_2_Cl_2_–MeOH) to obtain five fractions I–V. Fraction I (1.98 g) was further purified by CC (1.5 × 80 cm; 20 g of silica gel 230–400 mesh) eluting with CH_2_Cl_2_–MeOH (50:1 to 40:1) to afford compounds **2** (8 mg) and **3** (3 mg), respectively. Fraction II (1.86 g) resulting from CH_2_Cl_2_–MeOH (35:1 to 20:1) was chromatographed by CC (1.5 × 80 cm; 20 g of silica gel 230–400 mesh) using CH_2_Cl_2_–MeOH (40:1 to 15:1) to afford compound **4** (180 mg). Fraction III (2.73 g) resulting from CH_2_Cl_2_–MeOH (20:1 to 10:1) was chromatographed by CC (2.5 × 100 cm; 60 g of silica gel 230–400 mesh) to afford three sub-fractions IIIa–IIIc. Sub-fraction IIIa (0.88 g) was further purified by CC (1 × 25 cm; 40 g of silica gel 230–400 mesh) in gradient elution mixture solvent composed of CH_2_Cl_2_–MeOH (25:1 to 15:1) to afford compound **5** (10 mg). Sub-fraction IIIc (1.08 g) was further purified by silica gel column (1.5 × 80 cm; 20 g of silica gel 230–400 mesh) using CH_2_Cl_2_–MeOH (30:1 to 15:1) to produce a mixture of three components **6** (28 mg). Fraction IV (1.20 g) expected from CH_2_Cl_2_–MeOH (10:1 to 5:1) was rechromatographed (2 × 100 cm; 25 g of silica gel 230–400 mesh) using the solvent system CH_2_Cl_2_–MeOH (15/1 to 5/1) to give compound **1** (14 mg). Fraction V (4.42 g) afforded from CH_2_Cl_2_–MeOH (5:1 to 1:1) was chromatographed by CC over SiO_2_ eluted with CH_2_Cl_2_–MeOH (8:1 to 1:1) and purified by Sephadex LH-20 using pure MeOH as eluent to provide **7** (12 mg).

### (2R*)-2,3-Dihydrolophirone A (1a)

Orange amorphous powder; m.p. 265–269 °C; TLC Rf: 0.40 (CH_2_Cl_2_–MeOH; 10:1); IR (KBr) cm^−1^: 3444, 2970, 2854, 1710, 1652, 1603, 1572, 1071, 1054; ^1^H and ^13^C NMR spectral data (500 and 125 MHz, DMSO-*d_6_*), see Table 1; HR-ESI-MS *m/z*: 511.1396 [M–H]^−^ (calcd. C_30_H_24_O_8_-H: 511.1394); 535.1363 [M + Na]^+^ (calcd. C_30_H_24_O_8_Na^+^: 535.1369).

**Table 1.** ^1^H and ^13^C NMR spectroscopic data^a^ of compounds **1a** and **1b** (500 and 125 MHz in DMSO-*d_6_*) *δ* in ppm, with those of reference (compound **8**^c^).

	**1a**		**1b**		**8**^c^	
No.	*δ*_C_	*δ*_H_	*δ*_C_	*δ*_H_	*δ*_C_	*δ*_H_
1	71.4	3.79 (1H, –^b^) 3.39 (1H, –^b^)	155.6	8.06 (1H, s)	156.4	8.27 (1H, s)
2	49.4	2.76 (1H, m, *J* = 3.3, 6.9, 10.9)	125.4	–	122.1	–
3	190.6	–	174.5	–	175.4	–
4	115.8	–	115.7	–	117.1	–
5	128.9	7.60 (1H, d, *J* = 8.5)	128.9	7.60 (1H, d, *J* = 8.5)	128.2	7.91 (1H, d)
6	115.7	6.75 (1H, dd, *J* = 8.5; 2.3)	115.7	6.75 (1H, dd, *J* = 8.5; 2.3)	115.9	6.89 (1H, dd)
7	165.0	–	165.1	–	163.4	–
8	102.8	6.28 (1H, d, *J* = 2.3)	102.8	6.28 (1H, d, *J* = 2.3)	103.1	6.75 (1H, d)
9	159.0	–	159.0	–	158.5	–
10	49.1	4.04 (1H, dd, *J* = 12.4; 10.6)	50.7	4.98 (1H, –^b^)	43.9	6.12 (1H, d)
11	205.4	–	203.0	–	202.9	–
12	114.3	–	114.3	–	114.1	–
13	165.1	–	165.1		166.8	–
14	102.6	6.18 (1H, d, *J* = 2.3)	102.7	6.18 (1H, d, *J* = 2.3)	103.3	6.18 (1H, d)
15	165.6	–	165.6	–	166.8	–
16	108.7	6.48 (1H, dd, *J* = 8.5; 2.3)	108.7	6.48 (1H, dd, *J* = 8.5; 2.3)	109.0	6.42(1H, d)
17	132.9	7.39 (1H, d, *J* = 8.5)	132.9	7.39 (1H, d, *J* = 8.5)	134.4	8.32 (1H, d)
18	55.3	3.75 (1H, d, *J* = 12.4)	56.3	3.80 (1H, –^b^)	53.4	4.47 (1H, d)
19	132.9	–	133.2	–	135.6	–
20	130.6	7.28 (2H, d, *J* = 8.3)	130.6	7.28 (2H, d, *J* = 8.3)	129.4	7.24 (2H, d)
21	115.2	6.73 (2H, d, *J* = 8.3)	115.2	6.73 (2H, d, *J* = 8.3)	115.9	6.63 (2H, d)
22	156.0	–	156.0	–	156.5	–
23	115.2	6.73 (2H, d, *J* = 8.3)	115.2	6.73 (2H, d, *J* = 8.3)	115.9	6.63 (2H, d)
24	130.6	7.28 (2H, d, *J* = 8.3)	130.6	7.28 (2H, d, *J* = 8.3)	129.4	7.24 (2H, d)
25	133.1	–	133.1	–	134.5	–
26	129.5	7.29 (2H, d, *J* = 8.5)	129.5	7.29 (2H, d, *J* = 8.5)	130.0	7.23 (2H, d)
27	115.4	6.52 (2H, d, *J* = 8.5)	115.4	6.52 (2H, d, *J* = 8.5)	115.8	6.59 (2H, d)
28	157.6	–	157.6	–	156.4	–
29	115.4	6.52 (2H, d, *J* = 8.5)	115.4	6.52 (2H, d, *J* = 8.5)	115.8	6.59 (2H, d)
30	129.5	7.29 (2H, d, *J* = 8.5)	129.5	7.29 (2H, d, *J* = 8.5)	130.0	7.23 (2H, d)

^a^Assignments were confirmed by DEPT-135, HSQC, HMBC, ^1^H–^1^H COSY and NOESY experiments.

^b^Overlapping signals.

^c^Spectroscopic data recorded in acetone-*d_6_*.

### *Mycobacterium tuberculosis* strains

For the present study, the mycobacteria (*M. tuberculosis*) used were clinical isolated strains resistant to isoniazid and rifampicin codified AC45 and AC83, respectively (these strains were obtained from Sangmelima district’s Hospital in South Region of Cameroon). The genetic profile of the resistance has been carried out at Laboratory for Tuberculosis Research (Biotechnology Centre, University of Yaoundé I) through Line probe Assay method.

### Preparation and growth conditions of *M. tuberculosis*

The mycobacteria strain has been cultured at 37 °C for two weeks in Middlebrook 7H9 (Himedia, Mumbai, India) supplemented with 0.05% (v/v), 2% glycerol and 10% OADC (oleic acid–albumin–dextrose–catalase of Liofilchem s.r.l, Roseto degli Abruzzi, Italy). The optical density of 0.45–0.55 was measured using spectrophotometer at 550 nm to obtain a suspension of 1.5 × 10^8^ UFC/mL (Collins and Franzblau 1997).

### Preparation of extract and phytochemicals for antitubercular test

The activity of all phytochemicals (extract and pure compounds) against the aforementioned *M. tuberculosis* strains was tested using the microplate alamar blue assay (MABA) as described previously by Collins and Franzblau (1997) and Jiménez-Alleranes et al. (2003, 2007). In 96-well microplates, all wells received 100 µL of supplemented Middlebrook 7H9 broth, then working metabolites solutions (100 µL) were poured into the first well of each row, from which twofold dilution series were made through the microplate column. The test inoculum (100 µL) was added to all testing wells, as well as to the drug-free control wells. The final concentration of DMSO in wells was 7% v/v.

### Drug susceptibility testing of *M. tuberculosis*

#### Determination of minimum inhibitory concentration

MIC values were determined using the MABA; rifampicin and isoniazid were employed as references. The mycobacteria strain has been cultured at 37 °C for two weeks in Middlebrook 7H9 (Himedia, Mumbai, India) supplemented with 0.05% (v/v), 2% glycerol and 10% OADC (oleic acid–albumin–dextrose–catalase of Liofilchem s.r.l, Roseto degli Abruzzi, Italy). The 96-well plates received 100 μL of Middlebrook 7H9 broth and serial dilution of compounds was made directly on the plate with drug concentrations of 0.244–250 μg/mL and 5000 to 4.882 μg/mL for extracts. Plates were covered and sealed with parafilm and incubated at 37 °C for 14 days. Then, 40 μL of freshly prepared 1:1 mixture of alamar blue reagent and 7% Tween 80 (Himedia, Mumbai, India) was added to the plate and incubated for 24 h. A blue colour in the well was interpreted as no bacterial growth and pink colour was scored as growth. The MIC was defined as the lowest drug concentration, which prevented colour change from blue to pink. The results of antitubercular activity are depicted in Tables 2 and 3. Minimum inhibitory concentration (MIC) was determined by the broth micro-dilution method according to the guidelines of the Clinical and Laboratory Standards Institute (2011).

**Table 2.** MIC and MBC values of the methanol extract and the isolated compounds against *Mycobacterium tuberculosis* (AC45).

Plant species/compounds	MIC^a^ (µg/mL)	MBC^b^ (µg/mL)	MBC/MIC
*L. lanceolata*	312.5	625	2
Mixture of **1a** and **1b**	31.25	125	4
**2**	15.75	62.5	4
**4**	62.5	250	4
**5**	62.5	125	2
Mixture of **6a, 6b** and **6c**	250	250	1
**7**	62.5	125	2
**8**^c^	62.5	125	2
**RMP**	0.976	7.8125	8

RMP: rifampicin.

^a^Minimum inhibitory concentration.

^b^Minimum bactericidal concentration.

^c^Compound **8** (authentic sample available in our laboratory).

**Table 3.** MIC and MBC values of the methanol extract and the isolated compounds against *Mycobacterium tuberculosis* (AC83).

Plant species/compounds	MIC^a^ (µg/mL)	MBC^b^ (µg/mL)	MBC/MIC
*L. lanceolata*	1250	2500	2
Mixture of **1a** and **1b**	62.5	125	2
**2**	62.5	125	2
**4**	125	250	2
**5**	nd^d^	–	–
Mixture of **6a, 6b** and **6c**	250	–	–
**7**	125	250	2
**8**^c^	125	–	–
**INH**	3.904	15.625	4

INH: isoniazid.

^a^Minimum inhibitory concentration.

^b^Minimum bactericidal concentration.

^c^Compound **8** (authentic sample available in our laboratory).

^d^Not determined: nd (CMI >250 µg/mL).

#### Determination of minimum bactericidal concentration

The minimal bactericidal concentration (MBC) determination, 50 μL of each wells which concentration was ≥ MIC was sub-cultured in 150 μL of Mbk 7H9 medium and incubated at 37 °C for 10 days, then mycobacterial growth was carried out by addition of 40 μL of alamar blue. MBC was defined as the lowest concentration of extract at which no visible growth of the germ was observed. Minimum bactericidal concentration was determined by the broth micro-dilution method according to the guidelines of the CLSI 2011.

## Results and discussion

Crude MeOH extract from *Lophira lanceolata* was evaluated against two strains of *M. tuberculosis*. The strain AC45 of *M. tuberculosis* was more susceptible to MeOH root extract than AC83, with MIC values of 312.5 and 1250 µg/mL (Tables 2 and 3), respectively. Suggesting that the strain AC83 is more resistant than AC45. This extract was chromatographed on a column of silica gel eluted with CH_2_Cl_2_–MeOH in increasing polarity to afford compounds **1–7**. Compounds **2–7** were identified as known; stigmasterol (**2**) (Habib et al. 2007), ergosterol (**3**) (Feitosa et al. 2016), sitosterol-3-*O*-β-d-glucopyranoside (**4**) (Ngono Bikobo et al. 2015), lanceolin C (**5**) (Messanga et al. 1998), the mixture of campesterol-3-*O*-β-d-glucopyranoside (**6a**), sitosterol-3-*O*-β-D-glucopyranoside (**6b**), stigmasterol-3-*O*-β-d-glucopyranoside (**6c**) (Ngono Bikobo et al. 2014), isombamichalcone (**7**) (Ghogomu et al. 1989a, 1989b). Those structures were deduced from comparison of their spectral data with those reported in the literature.

**Figure 1.**
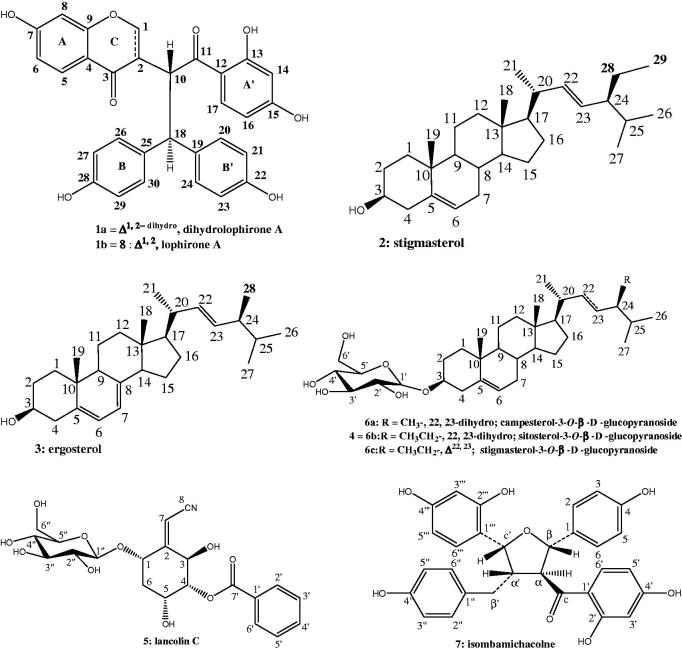
Constituents isolated from *Lophira lanceolata*.

**Figure 2.**
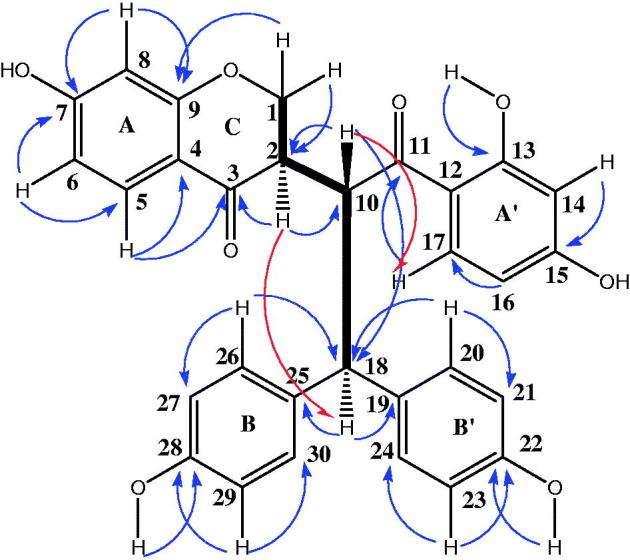
Selected HMBC 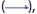 NOESY 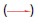 and COSY 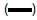 correlations for compound **1a**.

Compound **1** was obtained as an orange amorphous powder and a single peak in the UPLC profile. However, two sets of signals with almost the same intensities in the ^1^H NMR spectrum suggested that **1** was a mixture of two unseparated components, **1a** and **1b**, which were present in an approximate 2:1 ratio. This is strengthened by the HR-ESI-MS recorded in the negative and positive ion modes which exhibited different peaks at *m/z* 511.1396; 509.1238 [M–H]^−^, and 535.1363; 533.1305 [M + Na]^+^ corresponding to molecular formulas C_30_H_24_O_8_ and C_30_H_22_O_8_, respectively, suggesting that **1a** and **1b** had almost the same skeleton, with the presence of two additional hydrogen atoms for the first compound (see Supplemental data). Although **1a** and **1b** appeared to be homogenous based on TLC monitoring, thorough spectroscopic analyses were carried to determine the structure of these two unseparated compounds. The IR spectrum of **1** showed a broad hydroxyl absorption band at 3444 cm^−1^, signals at 1710 cm^−1^ (unconjugated carbonyl group), 1652 cm^−1^ (conjugated carbonyl groups), 1603 and 1572 cm^−1^ (aromatic rings) and 1071 cm*^−^*^1^ (C–*O*–C groups). The ^1^H NMR and ^1^H–^1^H COSY spectra of **1a** indicated the presence of two 1,2,4-trisubstituted benzene moieties at *δ*_H_ 6.28 (1H, d, *J* = 8.4 Hz, H-8), 6.75 (1H, dd, *J* = 2.3, 8.5 Hz, H-6) and 7.60 (1H, d, *J* = 8.5 Hz, H-5) (ring A). Other data at *δ*_H_ 6.18 (1H, d, *J* = 2.3 Hz, H-14), 6.48 (1H, dd, *J* = 2.3, 8.5 Hz, H-16) and 7.39 (2H, d, *J* = 8.5 Hz, H-17) suggested ring A′, when two 1,4-disubstitued benzene moieties could be identified at *δ*_H_ 6.52 (2H, d, *J* = 8.5 Hz, H-27/-29) and 7.29 (2H, d, *J* = 8.5 Hz, H-26/-30) (ring B); ring B′ appeared through signals at *δ*_H_ 6.73 (2H, d, *J* = 8.5 Hz, H-21/-23) and 7.28 (2H, d, *J* = 8.5 Hz, H-20/-24). In addition, the presence of a methine proton and a methylene group for compound **1a** was remarkable at *δ*_H_ 2.76 (1H, m, *J* = 3.3, 6.9, 10.9 Hz, H-2) and 3.39 and 3.79 (2H, m, H-1), respectively. Additionally, an AB system involved in *trans*-relationship was recognized at *δ*_H_ 4.04 (1H, d, *J* = 10.6, 12.4 Hz, H-10) and 3.75 (1H, d, *J* = 6.9, 12.4 Hz, H-18) as depicted in Figure 2. The ^13^C NMR of **1a** showed the resonances of 30 carbons consisting of 10 quaternary aromatic carbons at *δ*_C_ 114.3 (C-12), 115.8 (C-4), 130.9 (C-19), 133.1 (C-25), 156.0 (C-22), 157.6 (C-28), 159.0 (C-9), 165.0 (C-7), 165.1 (C-13) and 165.6 (C-15), along with two carbonyl carbons at *δ*_C_ 190.6 and 205.4, fourteen sp^2^ methine carbons (*δ*_C_ 102.6–132.9), two sp^3^ carbons at *δ*_C_ 71.5 (C-1) (methylene) and at *δ*_C_ 49.4 (C-2) (methine). Moreover, signals of two sp^3^ methine carbons at *δ*_H_ 49.1 (C-10) and 55.3 (C-18) indicated the presence of partial structure that was in accordance with ethyl group as shown in Figure 1. The cross-peaks of H-1 with H-2 and H-10 in the ^1^H–^1^H COSY spectrum indicated the presence of a partial structure that was consistent with a chromone derivative group. All these signals were in good agreement with those of the Lophirone A skeleton (Ghogomu et al. 1987). The complete assignment of all proton and carbon resonances was achieved after careful analysis of COSY, HSQC and HMBC techniques. The chemical structure of **1a** was established with the aid of the HMBC spectrum; the chromone moiety (ring AC) was assigned by the long range correlations between H-2 (*δ*_H_ 2.76) and C-1 (*δ*_C_ 71.5), C-3 (*δ*_C_ 190.6) and C-10 (*δ*_C_ 49.1). Additional correlations concerned H-1 (*δ*_H_ 3.39) and C-2 (*δ*_C_ 49.4), suggesting that the ring C of the chromone moiety was hydrogenated, and H-5 (*δ*_H_ 7.60) with C-3 (*δ*_C_ 190.6), C-4 (*δ*_C_ 115.8) and C-9 (*δ*_C_ 159.0) indicating the attachment of ring C to C-10. Further elements from HMBC predict correlations between H-17 and C-11 (*δ*_C_ 205.4), C-15 (*δ*_C_ 165.6), and between H-18 with the ketone carbonyl of C-11 (*δ*_C_ 205.6) corroborating the presence of an ethyl moiety in **1a**. This ethyl substructure was linked to C-18 of the molecule based on the HMBC correlations between H-20 (*δ*_H_ 7.28), H-26 (*δ*_H_ 7.29), with C-18 (*δ*_H_ 55.3) as shown in Figure 2. In addition, further elements from NOESY between H-10 (*δ*_H_ 4.04) and H-17 (*δ*_H_ 7.39) indicated the attachment of ethyl group to benzoyl moiety (ring A′). Moreover, HMBC correlation between an hydroxyl group at *δ*_H_ 12.6 with carbon at *δ*_C_ 165.1 (C-13), other hydroxyl groups at *δ*_H_ 9.48 and *δ*_H_ 9.07 correlating with carbons at *δ*_C_ 157.6 (C-28) and 156.0 (C-22) respectively indicated the attachment of these hydroxyls to ring A′, B and B′ separately. The relative configuration of carbon C-2 was determined from coupling constant of H-2 proton and from NOESY results (Figure 2). A 10.9 Hz value of this proton suggested the *trans*-relationships with H-10 (10.6 Hz). The NOESY correlation observed between H-2 (*δ*_H_ 2.76) and H-18 (*δ*_H_ 3.75) indicated the common orientation on the same side of the molecule for these protons. The structure of **1a** was therefore assigned as (2R*)-2,3-dihydrolophirone A.

The ^1^H and ^13^C NMR spectra of **1b** were very similar to those of **1a**. In the ^1^H NMR spectrum, signals due to two 1,2,4-trisubstituted benzene, two 1,4-disubstitued benzene, two methine protons involved in *trans*-relationships at *δ*_H_ 3.80 (1H, d, *J* = 12.0 Hz, H-18) and 4.98 (1H, d, *J* = 12.0 Hz, H-10), and one sp^2^ methine singlet at *δ*_H_ 8.06 (H-1) were evident, and these signals were in good agreement with those reported in literature (Ghogomu et al. 1987). The slight difference in the chemical shifts between H-18 for **1a** (*δ*_H_ 3.75) and **1b** (*δ*_H_ 3.80) might be explained by the dehydrogenation of chromone moiety for compound **1a**. However, ^13^C NMR spectrum showed significantly different chemical shift values from those of **1a** from chromone moiety. The appearance of carbons at *δ*_C_ 155.6 (C-1) and *δ*_C_ 125.4 (C-2) in **1b** instead of the methylene and methine carbons (*δ*_C_ 71.5, C-1 and 49.4, C-2) in **1a** was evident. The structure of **1b** was also determined through the interpretation of the HMBC spectrum, which showed long-range correlations from H-1 (*δ*_H_ 8.06) to C-2 (*δ*_C_ 125.4), C-3 (*δ*_C_ 174.5) and C-9 (*δ*_C_ 159.0) and from H-10 (*δ*_H_ 4.98) to C-2 (*δ*_C_ 125.4) and C-3 (*δ*_C_ 203.0). All these results were very similar to those of reported lophirone A. Furthermore, the NOESY experiment of compound **1b** showed cross-peaks from proton signal at *δ*_H_ 4.98 (H-10) to proton signal at *δ*_H_ 7.39 (H-17), indicating the attachment of the benzoyl moiety (nucleus A′) to methine carbon (C-10) of ethyl group as described for compound **1a**. Therefore, the structure of **1b** was identified as the known lophirone A.

In order to assess the biological effects of the compounds isolated from MeOH extract, antimycobacterial assays were performed. The antimycobacterial effect was assessed by testing compounds against two resistant strains of *M. tuberculosis*. Moreover, the methanol extract of *L. lanceolata* showed higher antimycobacterial activity in *M. tuberculosis* AC45 (MIC 312.5 µg/mL) than *M. tuberculosis* AC83 (MIC 1250 µg/mL) in the MABA assay, and it indicates that these results can be associated with differences in pathogenesis and virulence of the two strains or different pathogenic phenotypes between AC45 and AC83.

Although plants are an excellent source of diverse molecules, there is no plant based drug for the treatment of TB (Tiwari et al. 2013). The present study has brought out the antitubercular activities of compounds **1**, **2**, **4**, **5**, **6** and **7** against two clinical isolate strains of *M. tuberculosis* AC 45 and AC 83 in MABA assay. From these results (listed in Tables 2 and 3), compounds **1** and **2** exhibited the most potent antitubercular activity at MIC 31.25 and 15.75 µg/mL, respectively, against *M. tuberculosis* AC45. There are few studies describing the antimycobacterial activity of biflavonoids especially chalcone derivatives and steroids. In this respect, stigmasterol and the biflavonoids (dihydrolophirone A and lophirone A) can be considered as promising isolated compounds according to Cantrell et al. (2001). Compound **6** possessed antitubercular activity at MIC 250 µg/mL against the two tested strains and was considered inactive (Tables 2 and 3). In addition, compound **2** was more active than analogues (**4** and **6**) against *M. tuberculosis* suggesting an impact of polar substituents which showed a decreased inhibitory potency for antimycobacterial effect, implying an important role of the substitution patterns at C-3 (Figure 1). In assessing structure–activity relationships, the higher activity of compound **1** (MIC 31.25 µg/mL) compared to that of compound **8** (a pure sample of lophirone A, MIC 62.5 µg/mL) might be explained by a higher lipophilicity of the pair (**1a** and **1b**) which destabilizes the cytoplasmic membrane of microorganism, thereby reducing the pH gradient across the membrane and induces the cell death (Zengin and Baysal 2014). Nevertheless, the antimycobacterial activity of **1a** was not well evaluated because it appeared as mixture knowing that the position of hydroxyl group in phenolic ring is not recognized to strongly influence the degree of antibacterial activity (Shan et al. 2005), this is exemplified by the MIC values of compounds **7** and **8** at 62.5 µg/mL. However, according to Castellar et al. (2011), in all researches, no correlation on structure activity of flavonoids against mycobacteria could be drawn. Ergosterol (**3**) could not be tested due to its insufficient quantity. The antimycobacterial properties of some derivatives such as ergosterol-5,8-endoperoxide isolated from *Ajuga remota* are documented (Cantrell et al. 1999). The activity detected for **2** (MIC 15.75 µg/mL) and **4** (MIC 62.5 µg/mL) is twofold better than those previously reported (Thakur and Gothwal 2015). No antimycobacterial activity was reported earlier for the test compounds except for compound **4** which showed similar results against the resistant strain to isoniazid AC 45 (Evina et al. 2017; Tiam et al. 2017).

It was observed that all the compounds possessed very weak and/or no activity against *M. tuberculosis* AC83. Regarding the MBC values of both extracts tested (Tables 2 and 3), it seems that they could be similar to their MIC values against the tested organisms. These results also imply that all tested compounds exhibit bactericidal action against the studied strains (Peterson and Shanholtzer 1992).

Many plants of our continent are used locally in the treatment of TB, but their anti-tubercular properties have not been investigated due to the lack of appropriated material and scientific facilities. Therefore, it is imperative for African governments in general and in Sub-Sahara Africa in particular to make an effort to fund anti-tubercular drug research.

This study is the first report of antimycobacterial activity of constituents isolated from *L. lanceolata* against *M. tuberculosis*.

## Conclusions

The data illustrate that MeOH extract of the roots of *L. lanceolata* has exhibited less pronounced *in vitro* antimycobacterial activities and a number of active plant derived compounds belonging mainly to two chemical classes, steroid and bioflavonoid derivatives, exhibited good to moderate antimycobacterial activities. These bioactive phytochemicals can be used or utilized as promising candidate substances to develop new tools in antitubercular research. Additionally, there is a strong positive correlation between the antimycobacterial activity results and the ethnomedical/traditional usage on this plant against TB and TB-related diseases.

## Supplementary Material

Supplemental Material
