# Supplementary material for: Antitubercular evaluation of root extract and isolated phytochemicals from Lophira lanceolata against two resistant strains of Mycobacterium tuberculosis
Source: Pharm Biol. 2018 Jul 3;56(1):318–24. doi: 10.1080/13880209.2018.1476559 (PMC6130701; doi:10.1080/13880209.2018.1476559)
Supplement: Supplemental Material [file IPHB_A_1476559_SM6494.docx]

**Supplemental Material**

**Antitubercular evaluation of root extract and isolated phytochemicals from**

***Lophira lanceolata* against two resistant strains of *Mycobacterium tuberculosis***

**Jeanne Louise Nkot^a^, Dominique Serge Ngono Bikobo^a,e^*, Auguste Abouem A**

**Zintchem^a,b^, Norbert Mbabi Nyemeck II^a,c^, Esther Del Florence Moni Ndedi^d^, Patrick**

**Hervé Betote Diboué^d^, Dieudonné Emmanuel Pegnyemb^a^, Christian G. Bochet^e^, and**

**Ulrich Koert^c^**

^a^Department of Organic Chemistry, Faculty of Science, University of Yaoundé I, P.O Box

812, Yaoundé, Cameroon;

^b^Department of Chemistry, Higher Training College, University of Yaoundé I, P.O Box 47,

Yaoundé, Cameroon;

^c^Philipps-Universität Marburg, Faculty of Chemistry, Hans-Meerwein-Strasse, D-35032

Marburg, Germany;

^d^Department of Microbiology, Faculty of Science, University of Yaoundé I, P.O Box 812,

Yaoundé, Cameroon;

^e^Department Chemie, Universität Fribourg, CH du Musée 9, 1700 Fribourg, Switzerland

^*^Contact: Dominique Serge Ngono Bikobo

E-mail: [ngonosh@gmail.com](mailto:ngonosh@gmail.com)

Address: Department of Organic Chemistry, Faculty of Science, University of Yaoundé I, P.O Box812, Yaoundé, Cameroon

**Fig. S1 HR-**ESI-MS spectrum of compound **1 (negative mode)**

**Fig. S2 HR-**ESI-MS spectrum of compound **1 (Positive mode)**

**Fig. S3** ESI-MS spectrum of compound **1 (positive mode)**

**Fig. S4** ^1^H-NMR spectrum of compound **1**

**Fig. S5** ^13^C-NMR spectrum of compound **1**
